# Detection of astrocytic tau pathology facilitates recognition of chronic traumatic encephalopathy neuropathologic change

**DOI:** 10.1186/s40478-022-01353-4

**Published:** 2022-04-11

**Authors:** Kamar E. Ameen-Ali, Abigail Bretzin, Edward B. Lee, Rebecca Folkerth, Lili-Naz Hazrati, Diego Iacono, C. Dirk Keene, Julia Kofler, Gabor G. Kovacs, Amber Nolan, Daniel P. Perl, David S. Priemer, Douglas H. Smith, Douglas J. Wiebe, William Stewart, CONNECT-TBI Investigators, Safa Al-Sarraj, Etty Cortes, John Crary, Kristin Dams-O’Connor, Ramon Diaz-Arrastia, Jean-Pierre Dollé, Brian Edlow, Bruce Fischl, Col. Sidney Hinds, Victoria E. Johnson, Geoffrey Manley, David Meaney, David Okonkwo, Andrea L. C. Schneider, Julie Schneider, Claire Troakes, John Q. Trojanowski, Andre van der Kouwe, Kristine Yaffe

**Affiliations:** 1 Institute of Neuroscience and Psychology, University of Glasgow, Queen Elizabeth University Hospital, Glasgow, UK; 2 Department of Biostatistics, Epidemiology and Informatics, Perelman School of Medicine, University of Pennsylvania, Philadelphia, PA USA; 3 Department of Pathology and Laboratory Medicine, Perelman School of Medicine, University of Pennsylvania, Philadelphia, PA USA; 4 Office of Chief Medical Examiner, New York, NY USA; 5 Department of Forensic Medicine, New York University School of Medicine, New York, NY USA; 6 Department of Laboratory Medicine and Pathobiology, University of Toronto, Toronto, ON Canada; 7 Department of Pathology, The Hospital for Sick Children, Toronto, ON Canada; 8 Department of Defense/Uniformed Services, University Brain Tissue Repository and Neuropathology Program, Uniformed Services University, Bethesda, MD USA; 9 Department of Pathology, F. Edward Hébert School of Medicine, Uniformed Services University, Bethesda, MD USA; 10 Department of Neurology, F. Edward Hébert School of Medicine, Uniformed Services University, Bethesda, MD USA; 11 Neurodegeneration Disorders Clinic, National Institute of Neurological Disorders and Stroke, NINDS, NIH, Bethesda, MD USA; 12 The Henry M. Jackson Foundation for the Advancement of Military Medicine, Inc., Bethesda, MD USA; 13 Department of Laboratory Medicine and Pathology, University of Washington, Seattle, WA USA; 14 Department of Pathology, University of Pittsburgh School of Medicine, Pittsburgh, PA USA; 15 Tanz Centre for Research in Neurodegenerative Disease (CRND) and Department of Laboratory Medicine and Pathobiology, Krembil Discovery Tower, University of Toronto, 60 Leonard Ave, Toronto, ON Canada; 16 Laboratory Medicine Program and Krembil Brain Institute, University Health Network, Toronto, ON Canada; 17 Center for Brain Injury and Repair, Department of Neurosurgery, Perelman School of Medicine, University of Pennsylvania, Philadelphia, PA USA; 18 Department of Neuropathology, Laboratory Medicine Building, Elizabeth University Hospital, Glasgow, Queen UK; 19 Kings College London, London, UK; 20 Department of Pathology, Icahn School of Medicine at Mount Sinai, New York, NY USA; 21 Ronald M. Loeb Center for Alzheimer’s Disease, Departments of Neuroscience, Neurology and Genetics and Genomic Sciences, Icahn School of Medicine, New York, NY USA; 22 Department of Pathology, Fishberg Department of Neuroscience, Icahn School of Medicine at Mount Sinai, Friedman Brain Institute, New York, NY USA; 23 Department of Rehabilitation Medicine, Icahn School of Medicine at Mount Sinai, New York City, NY USA; 24 Department of Neurology, University of Pennsylvania Perelman School of Medicine, Philadelphia, PA USA; 25 Division of Neurocritical Care and Emergency Neurology, Massachusetts General Hospital, Boston, MA USA; 26 Athinoula A. Martinos Center for Biomedical Imaging, Department of Radiology, Massachusetts General Hospital & Harvard Medical School, Charlestown, MA USA; 27 Brain Tissue Repository & Neuropathology Core, Center for Neuroscience and Regenerative Medicine (CNRM), Uniformed Services University (USU), Bethesda, MD USA; 28 Chronic Effects of NeuroTrauma Consortium (CENC), Fort Detrick, MD USA; 29 Department of Neurological Surgery, University of California San Francisco, San Francisco, CA USA; 30 Brain and Spinal Injury Center, Zuckerberg San Francisco General Hospital, San Francisco, CA USA; 31 Department of Bioengineering, University of Pennsylvania, Philadelphia, PA USA; 32 Department of Neurological Surgery, University of Pittsburgh, Pittsburgh, PA USA; 33 University of Pennsylvania, Philadelphia, PA USA; 34 Department of Pathology, Rush University, Chicago, IL USA; 35 Department of Neurology, Epidemiology and Biostatistics, University of California San Francisco, San Francisco, CA USA

**Keywords:** Chronic traumatic encephalopathy, Traumatic brain injury, Neurodegeneration, Tau, Aging-related tau astrogliopathy

## Abstract

Traumatic brain injury (TBI) is associated with the development of a range of neurodegenerative pathologies, including chronic traumatic encephalopathy (CTE). Current consensus diagnostic criteria define the pathognomonic cortical lesion of CTE neuropathologic change (CTE-NC) as a patchy deposition of hyperphosphorylated tau in neurons, with or without glial tau in thorn-shaped astrocytes, typically towards the depths of sulci and clustered around small blood vessels. Nevertheless, although incorporated into consensus diagnostic criteria, the contribution of the individual cellular components to identification of CTE-NC has not been formally evaluated. To address this, from the Glasgow TBI Archive, cortical tissue blocks were selected from consecutive brain donations from contact sports athletes in which there was known to be either CTE-NC (n = 12) or Alzheimer’s disease neuropathologic change  (n = 4). From these tissue blocks, adjacent tissue sections were stained for tau antibodies selected to reveal either solely neuronal pathology (3R tau; GT-38) or mixed neuronal and astroglial pathologies (4R tau; PHF-1). These stained sections were then randomised and independently assessed by a panel of expert neuropathologists, blind to patient clinical history and primary antibody applied to each section, who were asked to record whether CTE-NC was present. Results demonstrate that, in sections stained for either 4R tau or PHF-1, consensus recognition of CTE-NC was high. In contrast, recognition of CTE-NC in sections stained for 3R tau or GT-38 was poor; in the former no better than chance. Our observations demonstrate that the presence of both neuronal and astroglial tau pathologies facilitates detection of CTE-NC, with its detection less consistent when neuronal tau pathology alone is visible. The combination of both glial and neuronal pathologies, therefore, may be required for detection of CTE-NC.

## Introduction

Increased risk of neurodegenerative disease has long been recognized following exposure to traumatic brain injury (TBI), with an estimated 3–10% of dementia in the community thought to be influenced by prior exposure to TBI [33, 43]. While a range of clinical syndromes and associated neuropathologies are described among late survivors of TBI [15, 22, 23, 26, 35, 47], there has been particular interest paid to the specific TBI associated pathology of chronic traumatic encephalopathy (CTE) [36, 45, 46]. Nevertheless, although the neuropathology of CTE was first described many decades ago, formal consensus criteria for its neuropathological evaluation and identification only emerged in the last decade as provisional consensus criteria [37], which have since been refined [4]. These criteria currently define the pathognomonic lesion of CTE neuropathologic change (CTE-NC) as a patchy cortical deposition of hyperphosphorylated tau (p-tau) in neurons, with or without glial tau in thorn-shaped astrocytes, typically clustered around blood vessels towards the depths of sulci [4]. Notably, however, although incorporated into consensus diagnostic criteria, the contribution of the individual cellular components to identification of CTE-NC has not been formally evaluated.

Following clinical descriptions of the punch drunk syndrome of former boxers early in the last century [34], the neuropathology of CTE, then described as dementia pugilistica, emerged several decades later in isolated case reports and short case series, again largely in former boxers [3, 7, 12, 17]. In these early accounts a range of neuropathological abnormalities was noted, including abundant neurofibrillary tangles [7, 12, 17] and amyloid-beta pathologies, the latter typically as diffuse plaques [41]. Of these, the presence of neurofibrillary tangles has attracted most attention, with early reports suggesting their distinctive cortical distribution might help to distinguish CTE from wider neurodegenerative pathologies. Specifically, neurofibrillary tangles in CTE were typically described as localised to more superficial cortical layers, often clustered around small blood vessels and with an impression that these may show particular concentration towards the depths of cortical sulci [12, 17]. Notably, while many of these observations have been incorporated into consensus criteria defining the pathognomonic lesion of CTE-NC, when subject to scrutiny, the impression of specific concentration of neurofibrillary tangles to sulcal depths appears less distinctive. Thus, using formal quantitative methodologies, neurofibrillary tangles in CTE show only a mild increase in density towards sulcal depths, mirroring that seen in Alzheimer’s disease neuropathologic change (ADNC) [1]. Further, although limited cryo-electron microscopy observations suggest the tau filament structure in CTE might differ from that of Alzheimer’s disease [9], the immunophenotype of the neuronal pathology in CTE appears indistinguishable from that of aging and ADNC, using currently available antibodies [2].

While p-tau immunoreactive neuronal profiles have long been recognised, only more recently have p-tau astroglial pathologies been documented in the context of aging and neurodegenerative disease [5, 27–30], with p-tau immunoreactive, thorn-shaped astrocytes recognised as a prominent component of the pathology of CTE-NC [20, 21, 25, 36, 38, 42]. Notably, although the thorn-shaped astrocytes of CTE-NC show comparable morphology and immunophenotype to those encountered in aging-related tau astrogliopathy (ARTAG) [2], quantitative assessment demonstrates this astrocytic pathology shows preferential concentrates at the depths of cortical sulci [1]. As such, while recent refinement to consensus neuropathological criteria proposes that the presence of p-tau immunoreactive astrocytes is no longer required for recognition of the pathognomonic lesion of CTE-NC [4], available evidence might suggest that their presence may still have some role in differentiating CTE from other neurodegenerative pathologies.

In this context, we hypothesise that, in routine diagnostic practice, the presence of both neuronal and astroglial tau pathologies is required for optimal detection of CTE-NC, with its detection less consistent when neuronal tau pathology alone is present. To this end, using antibodies that detect either neuronal tau pathology alone or that detect both neuronal and astroglial pathologies we performed blinded, multi-reviewer, unbiased assessments of cortical p-tau pathologies in material from former contact sports athletes either with known CTE-NC or with ADNC. Our observations suggest the presence of both neuronal and astroglial tau pathologies facilitates detection of CTE-NC, with its detection less consistent when neuronal tau pathology alone is visible. Further, in cases with overwhelming cortical p-tau pathologies, application of a combination of tau antibodies might enhance detection of CTE-NC.

## Methods

All cases were obtained from the Glasgow TBI Archive, Queen Elizabeth University Hospital, Glasgow, UK. Brain tissue samples were acquired at routine diagnostic autopsy, with approval for research tissue donation and use in research provided by the West of Scotland Research Ethics Committee (17/WS/0164) and the Greater Glasgow and Clyde Biorepository (Application Number 340). Donors for inclusion in this study were identified within the database of the Glasgow TBI Archive by an independent researcher not involved in reviews of the pathology. Donors were selected as consecutive research brain donations from former contact sports athletes (soccer n = 13; rugby union n = 2; rugby union and boxing n = 1) with neurodegenerative disease diagnoses in life, where the original diagnostic neuropathology evaluation documented the presence in cortical sections of either the pathognomonic pathology of CTE-NC (n = 12) or ADNC (n = 4) by current consensus criteria [4, 19, 37, 39]. Clinical, demographic and neuropathological information, including integrated clinicopathological diagnoses [32] are presented in Table 1.

**Table 1:** Case demographics

Case	Age at death	Sex	Sport exposure	PMI (days)	Pathology	CTE-NC Stage	Integrated CPC diagnosis
1	70s	M	Rugby	2	CTE-NC	low	AD
2	80s	M	Soccer	1	CTE-NC	low	AD
3	70s	M	Rugby	0.5	CTE.NC	high	CTE
4	70s	M	Soccer	8	CTE-NC	high	DLB
5	80s	M	Soccer	1	CTE-NC	high	PDD
6	70s	M	Soccer	3	CTE-NC	high	CTE
7	60s	M	Soccer	11	CTE-NC	high	VaD
8	80s	M	Soccer	3	CTE-NC	high	CTE
9	90s	M	Rugby/Boxing	5	CTE-NC	high	CTE
10	70s	M	Soccer	3	CTE-NC	high	CTE
11	60s	M	Soccer	11	CTE-NC	high	CTE
12	70s	M	Soccer	6	CTE-NC	high	CTE
13	70s	M	Soccer	7	ADNC	NA	PDD
14	70s	M	Soccer	2	ADNC	NA	AD
15	70s	M	Soccer	3	ADNC	NA	DLB
16	70s	M	Soccer	8	ADNC	NA	AD

AD, Alzheimer's disease; ADNC, AD neuropathologic change; CPC, clinicopathological conference; CTE, chronic traumatic encephalopathy; CTE-NC, CTE neuropathologic change; DLB, dementia with Lewy Bodies; NA, not applicable; PDD, Parkinson’s disease dementia; PMI, post-mortem interval; VaD, vascular dementia

### Immunohistochemistry

At the time of the original diagnostic autopsy, whole brains were immersion fixed in 10% formal saline for a minimum of two weeks, following which the specimens were examined, sampled using standardised techniques consistent with consensus protocols [4, 37] and processed to paraffin tissue blocks as previously described [13]. From each case a cortical tissue block documented by the original reporting pathologist (WS) during diagnostic evaluation as containing representative neuropathological features was selected from which consecutive sections were prepared and stained for a panel of antibodies to different tau species. Sections were cut at 8 μm using a rotary microtome (Leica Microsystems, Wetzlar, Germany) and mounted onto Superfrost Plus microscope slides (Cellpath, Powys, UK), before deparaffinisation and rehydration to water and immersion in 3% aqueous hydrogen peroxide for 15 min to quench endogenous peroxidase activity. After washing, microwave pressure cooker heat-mediated antigen retrieval was performed as optimized for each antibody using either 0.1 M Tris ethylenediaminetetraacetic acid (EDTA) buffer (pH8) or citrate buffer (pH6), with or without formic acid pre-treatment. Sections were then blocked for 30 min using normal horse serum (Vector Labs, Burlingame, CA, USA), before incubation in the primary antibody at 4 °C for 20 h. Specifically, a panel of tau antibodies was applied targeting either: phosphoepitope S404 (PHF-1; 1:1000; courtesy Dr P Davies); Alzheimer’s conformation dependent tau (GT-38; 1:1000; University of Pennsylvania); or 3 (RD3; 1:3000; Merck Millipore, Germany) or 4 (RD4; 1:400; Merck Millipore, Germany) microtubule-binding domain repeats. Following incubation in primary antibody, sections were rinsed before incubation in a biotinylated universal secondary antibody (Vector Labs, Burlingame, CA, USA) for 30 min at room temperature. Antibody binding was visualised using a 3,3’-diaminobenzidine (DAB) peroxidase substrate kit (Vector Labs, Burlingame, CA, USA). Sections were then counterstained with haematoxylin, followed by rinsing, dehydration and coverslipping. Sections from a known positive control for each antibody were stained in parallel with test sections, with omission of the primary antibody in one section to control for non-specific binding.

### Whole slide scanning and assessment

On completion of staining, all 64 stained section (16 cases, each stained with four different antibodies) were scanned with a 20 × objective on a Hamamatsu Nanozoomer 2.0-HT slide scanner and saved as NDPI files. File order was then randomised and each file assigned a unique identifying number such that scanned images from each case were neither consecutive in sequence or in number on the slide viewing portal. Slides were then collated into three sets, each of 21 or 22 slides, for distribution and scoring. Nine neuropathologists with experience in the neuropathological assessment of neurodegenerative disease and co-investigators on the Collaborative Neuropathology Network Characterising Outcomes of TBI (CONNECT-TBI) programme [44] participated in assessing these digital slide sets for pathology (RF, LNH, DI, CDK, JK, GGK, AN, DPP, DSP). Participating neuropathologists were blinded not only to case related information (such as demographics, original integrated diagnosis) for each slide, but also to the antibody used and the primary aims of study. Information provided to each participating neuropathologist at recruitment described this study as an exercise exploring consistency in reporting CTE-NC, with no information that the slide sets for review contained multiple consecutive sections from individual cases stained for a panel of tau antibodies.

To access each slide set participants received a Uniform Resource Locator (URL) weblink which directed them to a Research Electronic Data Capture (REDCap) portal wherein individual stained sections were presented in a series of consecutive pages, each requiring a response to be completed before advancing to the next stained section and response page. Each response page included a unique URL link to the appropriate stained section on the Hamamatsu server, together with the response questions. After review of each digital section, participating neuropathologists were asked:

1. *Would you classify this case as CTE? (select yes/no)*
2. *From 0–100%, how confident are you in your rating? (using slide bar, select rating from 0–100%)*

Instruction on criteria for CTE-NC recognition was provided with reference to the original published consensus [37]. Neuropathologists evaluated each slide set independently, at their own pace. Once responses had been submitted for a scanned tissue section, progress to the next was automatic, with no ability to navigate backwards to previous sections. Further, complete responses for each slide set were required before the URL linking to the next slide set was released. For each case and stain, the cut-off set for consensus required that 75% or more of reviewers were in agreement, in line with the Royal College of Pathologists guidance for a diagnostic External Quality Assessment scheme [11].

Following completion of data capture for the original three slide sets, refined consensus criteria were published by the National Institute of Neurological Disorders and Stroke (NINDS) consensus panel suggesting neuronal tau pathology alone would be sufficient for recognition of CTE-NC, rather than a requirement that both neuronal and astroglial pathology are present, as described in the original consensus criteria [4]. To accommodate this revision, a further slide set was circulated. This fourth slide set contained 21 sections which had been assessed in the previous three circulations, but which were re-anonymised and re-randomised. These 21 slides were selected on review of data from the original circulations 1 to 3 to include 3R and GT-38-stained sections from the five cases in which there was 100% consensus in recognition of CTE-NC and three cases returning the lowest consensus in recognition of CTE-NC in PHF-1-stained sections, with the remaining slides randomly selected PHF-1 stained sections from among the consensus recognized CTE-NC cases from the first review screening. The same REDCap evaluation process was employed, with the additional instruction to the participating neuropathologists to review this final slide set using updated consensus criteria. The neuropathologists remained blind to all demographic and diagnostic information. Again, reviewers were unaware that multiple sections were included from individual cases, that differing antibodies had been applied and that this supplemental slide set was derived from the initial circulations.

### Statistical analysis

Non-parametric receiver operating characteristics (ROC) analyses were used to evaluate accuracy of rating CTE-NC. The area under the ROC curve (AUC) was calculated to assess rater accuracy of the slide set overall and separately by antibody. AUC values were interpreted as 1.0 = perfect, > 0.9 = outstanding, 0.8–0.89 = excellent, 0.7–0.79 = acceptable, 0.51–0.69 = poor, 0.5 = no discrimination [14, 18]. AUC equivalence tests were used to assess whether rater accuracy varied by stain. We also estimated whether sensitivity and specificity could be increased if two stains were applied in parallel, where a positive result with either stain classifies the slide as “Yes-CTE-NC” [6]. All analyses were performed using Stata/MP 16.1 (StataCorp LLC), with *p*-value less than 0.05 considered to indicate statistical significance.

## Results

### Identification of CTE-NC is influenced by the primary antibody applied

**Fig. 1:**
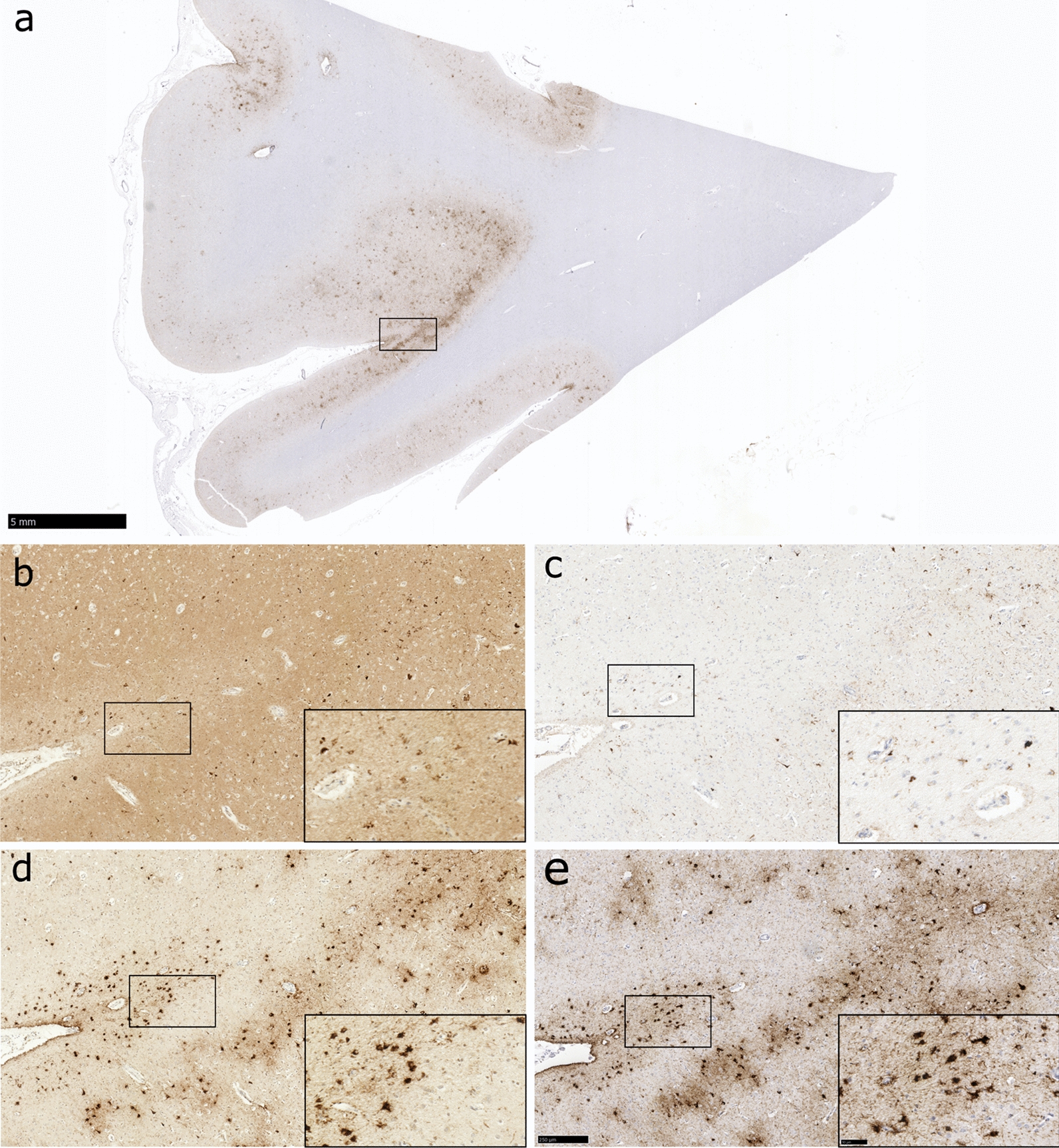
Representative images of cortical tau pathology in a male, 80-year-old former soccer player, with known high-stage CTE-NC (Case 5), stained for various tau antibodies**.** In sections stained for either PHF-1 (**a, e**) or 4R tau (**d**) staining of both neuronal and astroglial profiles is present in a patchy distribution, with concentration to sulcal depths consistent with the pathognomonic lesion of CTE-NC defined in original consensus criteria. In contrast, adjacent sections from the same case stained for either 3R tau **(b**) or GT-38 (**c**) show only neuronal pathologies. Scale bars: a, 5 mm; b-e main panels, 250 µm; b-e insets 50 µm

**Fig. 2:**
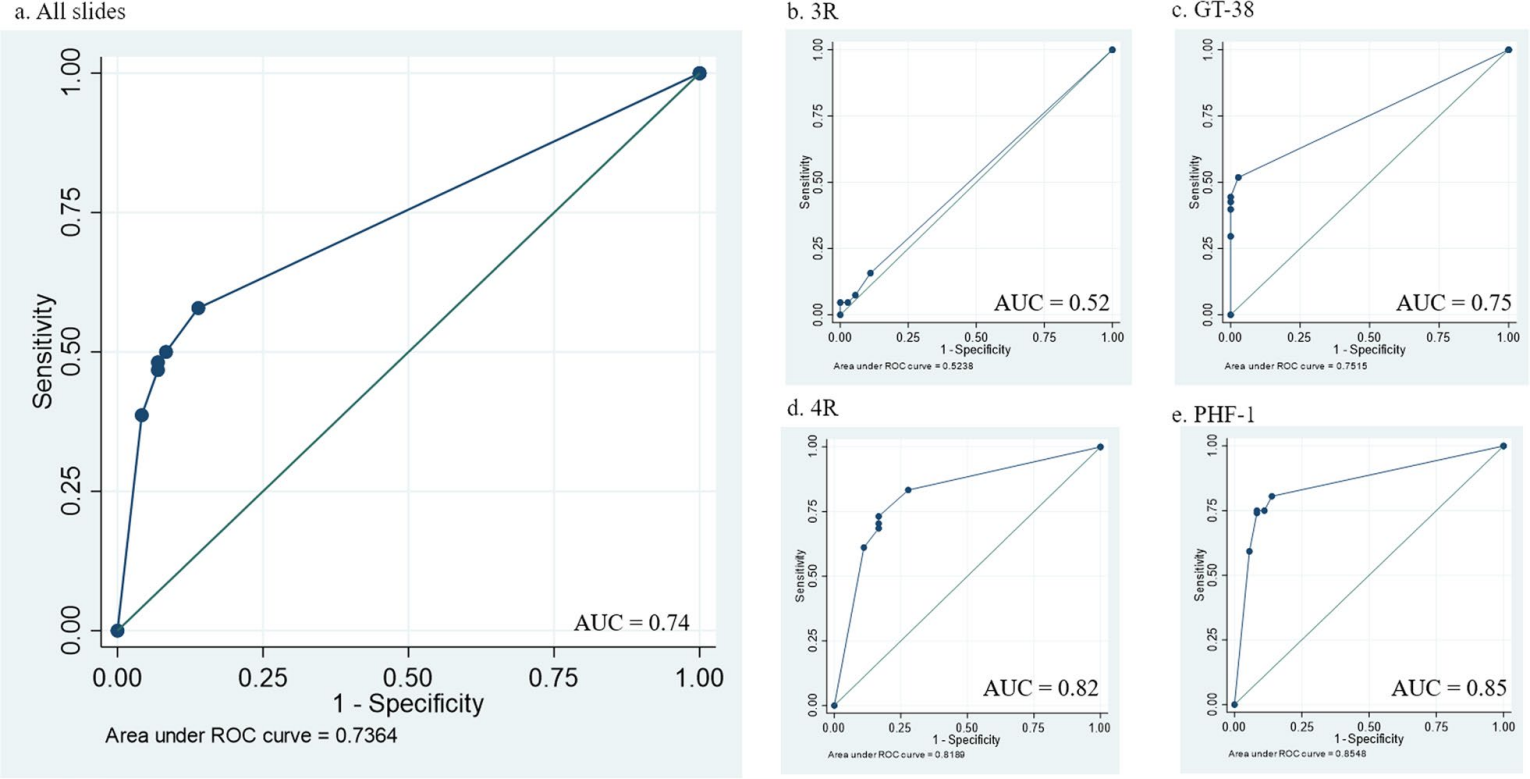
Receiver operating characteristic (ROC) curves summering the performance of each of the primary antibodies employed

Consistent with previous reports, sections stained with the tau antibodies employed revealed varying patterns of staining. Specifically, sections stained for PHF-1 or 4R tau typically revealed both immunoreactive neuronal and astroglial profiles, where present. In contrast, adjacent sections stained for either GT-38 or 3R tau revealed solely immunoreactive neuronal profiles (Fig. 1). Overall, using original consensus criteria, consensus recognition of CTE-NC as either absent or present was high among reviewers (area under the curve [AUC] 0.74; 95% confidence interval [CI] 0.70 to 0.77), although this varied depending on the primary antibody used. Reflecting this, consensus recognition of CTE-NC absent or present was highest in sections stained for PHF-1 (AUC 0.85; 95% CI 0.79 to 0.92) and lowest in sections stained for 3R tau (AUC 0.52; 95% CI 0.46 to 0.59) (Fig. 2; Table 2). Indeed, where CTE-NC was present, this was recognized in just 5% of sections stained for 3R tau, compared to 75% of sections stained for PHF-1 (Table 3).

**Table 2:** Performance of each tau antibody in consensus reporting of CTE-NC as absent or present

	AUC (95% CI)	Sensitivity %	Specificity %	PPV %	NPV %
All sections	0.74 (0.70–0.77)	57.9	86.1	92.6	40.5
PHF-1	0.85 (0.79–0.92)	80.6	86.1	94.6	59.6
4R	0.82 (0.74–0.89)	73.0	83.0	92.9	50.8
GT-38	0.75 (0.70–0.80)	51.9	97.2	98.2	40.2
3R	0.52 (0.46–0.59)	15.7	88.9	81.0	26.0

AUC = area under the curve; PPV = positive predictive value; NPV = negative predictive value; GT38 vs. 4R, *p* = 0.140; GT38 vs. PHF-1, *p* < 0.005; 4R vs. PHF-1*, p* = 0.375; 3R vs. PHF-1, *p* < 0.001

**Table 3:** Proportion of reviewers reporting CTE-NC for each slide and primary antibody

	Tau antibody			
Case No	3R	GT-38	4R	PHF-1
All sections	28%	57%	74%	79%
*CTE-NC*				
All sections	5%	43%	70%	75%
1	0	33%	56%	44%
2	0	56%	78%	89%
3	22%	0	100%	11%
4	11%	22%	78%	78%
5	0	44%	100%	100%
6	11%	33%	67%	100%
7	0	78%	44%	100%
8	0	67%	44%	67%
9	0	11%	56%	100%
10	0	78%	100%	89%
11	11%	89%	100%	100%
12	0	0	22%	22%
*ADNC*				
All sections	3%	0	17%	8%
13	0	0	0	0
14	0	0	56%	11%
15	0	0	11%	11%
16	11%	0	0%	11%

**Fig. 3:**
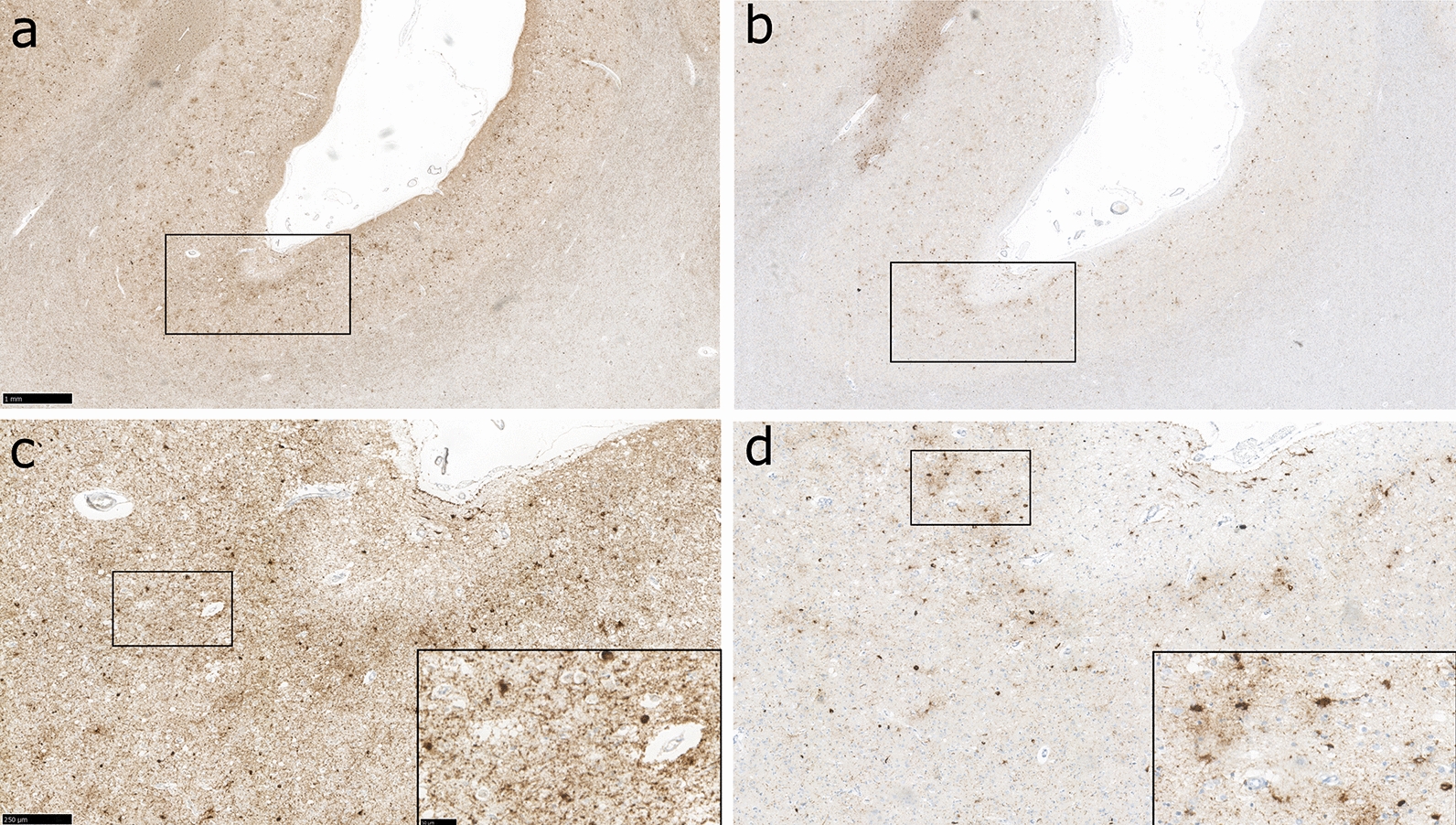
Representative images of cortical tau pathology in Case 3, a 73-year-old male, former rugby player, with known high-stage CTE-NC from the original diagnostic evaluation. In the section stained for PHF-1 extensive, confluent cortical staining is present within neuronal and astrocytic profiles with no particular cortical distribution discernible (**a, c**). In contrast, in an adjacent section stained for 4R tau, patchy cortical staining is evident, showing concentration to the depths of cortical sulci, consistent with the pathognomonic lesion of CTE-NC (**b, d**). Scale bars: a-b 1 mm; c-d main panels, 250 µm; c-d insets, 50 µm

Using original consensus criteria, although recognition of pathology was highest among sections stained for PHF-1, nevertheless, there remained 4 cases with known CTE-NC among which no consensus agreement was achieved in sections stained using this antibody, with detection rates varying between 11 and 67% in these examples (Table 3). Intriguingly, in the section stained for PHF-1 with lowest detection of CTE-NC (Case 3), while this pathology was only recognised by 1 of 9 (11%) of the reviewers, there was complete consensus on the presence of CTE-NC in the adjacent section from this case stained for 4R. Review of the sections in this case revealed extensive PHF-1-immunoreactive neurofibrillary tangles, neurites and thorn-shaped astrocytes at the sulcal depth, producing a near confluent band of staining. In contrast, in the adjacent 4R section a patchier staining pattern was evident, consistent with the original consensus description of CTE-NC (Fig. 3). In the remaining three cases for which consensus recognition of CTE-NC was not achieved, these showed extensive tau pathologies at the sulcal depths, with no improvement in consensus recognition of CTE-NC in adjacent sections stained for the remaining tau antibodies.

### Revised consensus criteria do not improve consensus in CTE-NC recognition

Immediately following primary section reviews and data collection, revised consensus criteria for the neuropathological assessment of CTE were published, placing greater emphasis on neuronal p-tau pathology, “with or without glial tau in thorn-shaped astrocytes” as sufficient for recognition of the pathognomonic lesion of CTE-NC [4]. Review of a supplemental slide set containing representative stained sections which had been assessed in the primary reviews, but under instruction to apply revised criteria for CTE-NC, resulted in no improvement in recognition of diagnostic pathology, either in neuronal specific or broader primary antibody preparations. Specifically, in sections stained with 3R, GT38, or PHF-1 overall recognition of CTE-NC did not differ between primary and secondary reviews (*p* = 0.766) (Table 4).

**Table 4:** Proportion of reviewers reporting CTE-NC applying original then revised NINDS consensus criteria to the same stained section

Case No	3R		GT-38		PHF-1	
	Primary review	Secondary review	Primary review	Secondary review	Primary review	Secondary review
1	0	11%	33%	44%	–	–
2	–	–	–	–	89%	67%
3	22%	22%	0	11%	–	–
4	–	–	–	–	78%	89%
5	0	11%	44%	11%	–	–
6	11%	22%	33%	33%	100%	100%
7	0	11%	78%	33%	–	–
8	–	–	–	–	67%	78%
9	0	0	11%	11%	–	–
10	–	–	–	–	89%	100%
11	11%	33%	89%	89%	–	–
12	0	0	0	0	–	–

## Discussion

Herein we present an analysis of unbiased, blinded reviews of cortical tau pathologies in chronic traumatic encephalopathy neuropathologic change (CTE-NC) employing a panel of antibodies revealing either solely neuronal or mixed neuronal and astroglial pathologies. In so doing, observations in our cohort demonstrate that recognition of the pathognomonic cortical pathology of CTE-NC is optimised by visualisation of both neuronal and astroglial tau pathologies. Specifically, in cortical tissue sections stained with antibodies revealing mixed neuronal and astroglial tau pathologies (PHF-1 or 4R tau), accurate consensus recognition of CTE-NC was high among expert neuropathology reviewers. In contrast, where adjacent sections from the same cases were stained for antibodies revealing solely neuronal tau pathology (GT-38 or 3R tau), accurate recognition of CTE-NC was considerably impaired; in the case of 3R tau, it was no better than chance. Importantly, these findings remained when refined NINDS criteria for neuropathological recognition of CTE-NC were employed, suggesting neuronal tau pathology alone might be insufficient in defining the pathognomonic lesion.

While descriptions of the pathology of CTE first emerged over half a century ago in reports of dementia pugilistica of boxers [7], only in the last decade have consensus criteria for the neuropathological evaluation of cases with suspected CTE and its recognition emerged [4, 37]. In the first iteration of these consensus criteria the contributing pathologists reviewed 10 cases with known high-stage CTE-NC selected from a single archive’s holdings, alongside 15 cases of wider tauopathies. Following review and discussion, the panel defined the pathognomonic lesion of CTE-NC as consisting of *‘p-tau aggregates in neurons, astrocytes, and cell processes around small vessels in an irregular pattern at the depths of cortical sulci’* [37]. Thereafter, in a second consensus review process the panel examined a further 16 cases with low (n = 4; two of which after consensus review were deemed to show no diagnostic pathology) or high (n = 12) stage CTE-NC, alongside 3 of the CTE-NC cases assessed in the original consensus review and 10 cases with wider, non-CTE-NC pathologies [4]. Following this second review process, the expert panel recommended the criteria for recognition of the pathognomonic cortical lesion of CTE-NC be refined, with the requirement for p-tau pathology in both neurons and astrocytes modified to place greater emphasis on the presence of p-tau in neurons alone being sufficient, ‘*with or without thorn shaped astrocytes*’ [4]. This refinement to the criteria for recognition of the pathognomonic lesion of CTE-NC, therefore, implied that a diagnosis can be made in the absence of p-tau immunoreactive astrocytes. Notably, however, both these consensus review processes employed subjective, qualitative methodologies, with review of pre-selected cases from a single archive’s wider holdings. Employing unbiased, blinded review by multiple expert neuropathologists, some of whom also participated in the NINDS consensus review panel, of consecutive cases donated to the Glasgow TBI Archive, our data show that in preparations where only neuronal p-tau profiles were stained, recognition of CTE-NC was impaired. In contrast, where both neuronal and astroglial tau pathologies were revealed, consensus recognition of CTE-NC was highest.

Various approaches for staging of CTE-NC have been proposed, although these have largely either not been subject to independent evaluation [40] or have failed to perform under consensus review [4, 36]. Thus, at present, the working recommendation is that a dichotomous approach to staging is adopted, with CTE-NC reported as either low- or high-stage disease [4]. Notably, just 2 cases in this current study and a further 2 cases in the NINDS consensus reviews were considered low-stage disease. Of the low-stage cases evaluated in this study, one of these (Case 2) showed clear consensus for CTE-NC in stains for 4R or PHF-1 but failed to be recognised as such in sections stained for 3R or GT-38. The second low-stage case (Case 1) failed to achieve consensus for CTE-NC in any of the stained sections. Although just two case observations, these data might be interpreted as suggesting that even in low stage disease, the presence of astroglial pathology aids recognition of CTE-NC. Nevertheless, it must be acknowledged that current published evaluations of CTE-NC are biased towards higher stage disease, with a total of just 4 low-stage cases reviewed across this study and the NINDS consensus processes. As such, it remains possible that with greater case experience, low-stage CTE-NC might still be defined as localised to solely neuronal or, conceivably, solely astroglial profiles.

The current study supports the importance of selection of an appropriate tau antibody to reveal a broad spectrum of pathology in the evaluation of CTE-NC. Previous work has shown that neuronal and astroglial tau pathologies in CTE-NC echo tau isoforms and immunophenotypes encountered in aging and Alzheimer’s disease (AD) [2]. Specifically, thorn-shaped astrocytes of CTE-NC are comprised solely of 4R tau and show similar post-translational modifications to those found in aging related tau astrogliopathy (ARTAG), while neuronal profiles echo the tau phenotypes found in primary age-related tauopathy (PART) and AD [2]. Notably, however, although subjective assessments of these pathologies in CTE-NC are widely reported as showing both neuronal and astroglial pathologies localised to the sulcal depths, including those of consensus reviews [4, 37], formal quantitative evaluation demonstrates primarily the astroglial profiles show specific concentration at this site, with the neuronal pathology showing only limited sulcal concentration, similar to that of AD [1]. Taken together with our current observations, these studies suggest that while neuronal p-tau in CTE-NC might be indistinguishable from that of aging and AD, both in sulcal distribution and immunophenotype using currently available antibodies for diagnostic practice, the astroglial pathology of CTE-NC shows distinctive cortical distribution. Therefore, the constant presence of astrocytic tau pathology in CTE-NC in a distinctive pattern and distribution supports its inclusion as required (i.e. pathognomonic) pathology alongside neuronal tau to facilitate the neuropathological diagnosis of CTE-NC.

The contribution of thorn-shaped astrocytes to recognition of CTE-NC revealed in this study raises the intriguing potential that, rather than incidental pathology of limited importance, the observed astrocytic p-tau pathology might be both specific to prior exposure to TBI and might provide evidence of an underlying, driving neurodegenerative process. Notably, early descriptions of ARTAG documented pathology in the medial temporal lobe, with more recent reports noting a resemblance of ARTAG to CTE-NC in some cases [30]. Importantly, thorn-shaped astrocytes in the depths of cortical sulci are rare in normal ageing [10] but can be seen in Guam amyotrophic/parkinsonism-dementia complex [29]. The distribution of ARTAG in many of these descriptions raises the possibility of underlying CSF- or blood–brain barrier disruption driving the observed pathology [28, 30]. Intriguingly, evidence of extensive, widespread blood–brain barrier (BBB) disruption has been observed following single moderate or severe TBI, which can persist even many years after injury [16]. Similarly, evidence of widespread BBB disruption has also been reported after repetitive mild TBI in humans [8], and as a pathological consequence of mild TBI in experimental models [24]. Mathematical modelling indicates that a local (i.e. mechanical) inducing factor might contribute to the development of subpial thorn-shaped astrocytes in cortical areas [30, 31]. On one hand, these observations suggest that BBB dysfunction is a common event in the pathogenesis of astroglial pathologies of CTE-NC and ARTAG, while on the other hand it might suggest that local mechanical factors contribute to the focal cortical ARTAG of aging or other conditions showing overlap with the pathogenesis of CTE.

In summary, our data demonstrate the presence of both neuronal and astroglial tau pathologies facilitates recognition of CTE-NC, with its detection less consistent when neuronal tau pathology alone is visible. The combination of both glial and neuronal pathologies, therefore, may be required for detection of CTE-NC. In addition, in cases where there is overwhelming cortical p-tau pathology, application of the 4R tau isoform antibody might aid in revealing CTE-NC. While further studies are required to continue the process of refinement of our understanding of the specific pathologies of CTE, including those of earlier stages in disease, this work further underlines the value of unbiased, blinded review and analysis over subjective methodologies in evaluating patterns of disease.

## Disclosure

The information, conclusion and opinions expressed herein do not necessarily represent the official position or policy of, nor should any official endorsement be inferred on the part of, the Uniformed Services University of the Health Sciences, the US Department of Defense, the Veterans Administration, the Henry M. Jackson Foundation for the Advancement of Military Medicine, Inc or any other US Government agency.

## Data Availability

The datasets used and/or analysed during the current study available from the corresponding author on reasonable request.

## Abbreviations

AD: Alzheimer’s disease
ADNC: Alzheimer’s disease neuropathologic change
ARTAG: Aging related tau astrogliopathy
AUC: Area under the curve
BBB: Blood–brain barrier
CONNECT-TBI: Collaborative Neuropathology Network Characterising Outcomes of TBI
CTE: Chronic traumatic encephalopathy
CTE-NC: Chronic traumatic encephalopathy neuropathologic change
DAB: 3,3’-Diaminobenzidine
EDTA: Ethylenediaminetetraacetic acid
NINDS: National Institute of Neurological Disorders and Stroke
PART: Primary age-related tauopathy
P-tau: Hyperphosphorylated tau
REDCap: Research Electronic Data Capture
ROC: Receiver operating characteristics
TBI: Traumatic brain injury
URL: Uniform resource locator
3R: 3 Microtubule-binding domain repeats
4R: 4 Microtubule-binding domain repeats
